# Developing a comprehensive BACnet attack dataset: A step towards improved cybersecurity in building automation systems

**DOI:** 10.1016/j.dib.2024.111192

**Published:** 2024-12-03

**Authors:** Seyed Amirhossein Moosavi, Mojtaba Asgari, Seyed Reza Kamel

**Affiliations:** Department of Computer Engineering, Mashhad Branch, Islamic Azad University, Mashhad, Iran

**Keywords:** BACnet, Building management system, BMS, Attack detection, Network security, SCADA, Cyber-physical system, CPS

## Abstract

With the development of smart buildings, the risks of cyber-attacks against them have also increased. One of the popular and evolving protocols used for communication between devices in smart buildings, especially HVAC systems, is the BACnet protocol. Machine learning algorithms and neural networks require datasets of normal traffic and real attacks to develop intrusion detection (IDS) and prevention (IPS) systems that can detect anomalies and prevent attacks. Real traffic datasets for these networks are often unavailable due to confidentiality reasons. To address this, we propose a framework that uses existing real datasets and converts them into BACnet protocol network traffic with detailed network behaviour. In this method, a virtual machine is prepared for each controller based on real scenarios, and by creating a simulator for the controller on the virtual machine, real data previously collected under real conditions from existing datasets is injected into the network with the same date and time during the simulation. We performed three types of attacks, including Falsifying, Modifying, and covert channel attacks on the network. For covert channel attacks, the message was modelled in three forms: Plain text, hashed using SHA3-256, and encrypted using AES-256. Network traffic was recorded using Wireshark software in pcap format. The advantage of the generated dataset is that since we used real data, the data behaviour aligns with real conditions.

Specifications Table

**Table:** 

Subject	Cryptography and Cybersecurity
Specific subject area	Cybersecurity of Building Automation System and Supervisory Control and Data Acquisition System
Type of data	Network Traffic (pcap)
Data collection	For the BACnet protocol traffic dataset, data from the Building and Energy Management System of Tampines College Global Campus in Southeast Asia, Singapore, was used. We simulated controllers based on the scenario in Fig. 2 and captured normal traffic using Wireshark in pcap format. Then, we performed Falsifying, Modifying, and covert channel attacks and generated related datasets by recording the network traffic
Data source location	Building and Energy Management System, Tampines College Global Campus, Southeast Asia, Singapore [1].
Data accessibility	Repository name: **kaggle** Data identification number: Direct URL to data: https://www.kaggle.com/datasets/78eb45aeaac481853135d90738672111e46fe2a4ce653573e8856fc920f3da68 Instructions for accessing these data: If you do not have a Kaggle account, click “Sign Up” to create a free account. If you already have an account, click “Log In” and enter your credentials. Once logged in, navigate back to the dataset page using the provided URL and click the “Download” button to download the dataset files. If prompted, agree to the Kaggle terms and conditions for data usage
Related research article	Miller, C., Nagy, Z., & Schlueter, A. (2014). A seed dataset for a public, temporal data repository for energy informatics research on commercial building performance. Conference Paper. DOI: 10.13140/RG.2.1.4620.8485

## 1 Value of the Data

• Due to the development of Building Automation Systems (BAS) and the widespread use of the Building Automation and Control network (BACnet) protocol in these systems, and the lack of datasets related to this protocol, especially labeled attack datasets, this dataset is useful for research related to anomaly detection in BACnet protocol network traffic.
• This dataset includes traffic from Falsifying, Modifying, and covert channel attacks, providing a diverse set of attacks.
• This dataset can be used to develop Intrusion Detection Systems (IDS) and Intrusion Prevention Systems (IPS) for the BACnet protocol.
• Simulated attack scenarios in BAS, such as data spoofing, modification, and covert channel attacks, enhance the realism of the dataset, providing practical insights for improving the security of these systems. These scenarios enable researchers to refine intrusion detection and anomaly detection algorithms, ultimately strengthening the resilience of smart building networks against cyber threats.
• This dataset and framework can support advancements in fault detection and cryptographic security within BAS. By combining real data with simulated attack scenarios, the dataset provides a practical foundation for testing fault detection algorithms and evaluating cryptographic methods that enhance BACnet communication security and data integrity in smart building environments.

## 2 Background

According to the compound annual growth rate (CAGR), the annual growth rate of the smart buildings market between 2023 and 2030 is approximately 26.5 % [2]. This high growth rate indicates the widespread and growing adoption of smart buildings, leading to the development of internal communication networks within buildings and their connection to the internet. The increasing smart building development raises the vulnerability and threats caused by cybercrimes in daily life [3]. One of the popular communication protocols in smart buildings is the BACnet (Building Automation and Control Networks) protocol, which was specifically introduced by the American Society of Heating, Refrigerating, and Air-Conditioning Engineers (ASHRAE) for building control systems such as HVAC, cooling and heating systems, fire alarm and extinguishing systems, and other building equipment [4]. Given the growing use of this protocol, studying its vulnerabilities and countermeasures against potential threats through this protocol is of great importance. Part of the actions related to the development of intrusion detection systems (IDS) and intrusion prevention systems (IPS) requires the use of machine learning, and what is crucial for using this method is the existence of a reliable dataset [5]. There is a shortage of suitable BACnet network traffic datasets for using learning methods for anomaly detection and intrusion detection [6]. Therefore, in this article, we not only propose a framework for generating the necessary datasets but also provide a suitable dataset for conducting anomaly detection and intrusion detection studies. The proposed framework uses existing smart building data and converts them into BACnet protocol network traffic, offering real-like data without noise, unlike simulation methods.

Vincent et al. proposed a method based on Graph Convolutional Networks (GCN) for detecting attacks in cyber-physical systems (CPS). They found that the input data in modelling are of great importance because proper analysis enables the detection and prevention of information attacks [7].

Arat et al. proposed a vulnerability and risk assessment method for critical systems enabled by IIoT, focusing on reducing risk factors and vulnerable structures. They emphasized the use of real datasets in experiments and evaluations to ensure the reality and reliability of the proposed methods [8].

Pinto et al. in their article stated that for effective learning in detecting cyber-attacks in distributed smart networks like IoTs, the training datasets must include representative samples of various attack events with different patterns. They find this challenging because generating such datasets is time-consuming and costly, and sometimes the attacks that some supervised machine learning algorithms can detect do not occur in real environments with normal datasets [9].

Lemay et al. provided a dataset for normal traffic and covert channel attack traffic in a SCADA network with the Modbus protocol. They identified four main challenges of datasets related to SCADA networks: the unavailability of data due to security issues, injection of malicious traffic, the impact of physical conditions on the data, and their labelling. They produced their dataset using simulation tools, but due to the lack of real data, they seem to have been unsuccessful in addressing the third challenge, which is the impact of physical conditions on the data [10].

Elnour et al. used simulation tools to create normal and attack data for the HVAC system of a simulated building. Due to the use of simulation tools, their provided dataset has challenges such as not reflecting physical conditions and noise in the dataset, not providing the dataset as network traffic, and the absence of a specific protocol in the traffic. Furthermore, covert channel attacks that impact the timing of packet transmission were not implementable [11].

Gaggero et al. introduced the ICS-ADD dataset, designed to simulate various cyber-attacks on industrial control systems (ICS) in smart industrial environments. This dataset, based entirely on a testbed with key ICS components such as SCADA, PLC, firewall, and Network Intrusion Detection Systems (NIDS), includes a variety of attack types, including Denial of Service (DoS), Man-in-the-Middle (MITM), and False Data Injection, capturing security events with tools like OSSIM and Suricata. However, relying on simulated data presents certain limitations, such as a lack of real-world physical conditions and reduced variability in network traffic, which can impact the dataset's practical applicability in real environments [12].

De Keersmaeker et al., in *A Survey of Public IoT Datasets for Network Security Research*, review 74 public datasets, categorizing them by characteristics such as traffic type, data content, scale, collection methodology, and attack scenario. While the survey includes datasets generated via simulations, testbeds, and real-world network captures, most of them rely heavily on synthetic or testbed data. Additionally, many of the surveyed datasets focus on IoT protocols commonly used in smart homes and smart cities, such as MQTT and CoAP, rather than protocols specific to Building Automation Systems (BAS), like BACnet. This focus limits their applicability to environments where specific protocols like BACnet are essential for securely managing building infrastructure, particularly against threats such as data spoofing, data modification, and covert channel attacks [13].

In the field of cybersecurity for smart buildings and industrial control systems, numerous studies emphasize the importance of reliable datasets for enhancing intrusion detection and anomaly detection capabilities. The primary similarity between our work and previous studies is the shared focus on leveraging machine learning and the need for representative data to support accurate security analysis. However, most existing datasets rely heavily on simulated or testbed data, which often fail to capture the physical conditions and environmental variability of real-world settings.

In contrast, our work centers on creating a dataset based on real data from smart building environments, offering a more accurate reflection of practical conditions. Unlike many datasets that focus on industrial protocols and general IoT protocols, our study specifically addresses attack scenarios unique to the BACnet protocol in Building Automation Systems (BAS). These distinctions make our dataset a practical and valuable resource tailored to the security needs of smart building infrastructures.

## 3 Data Description

Two folders are uploaded on kaggle. The “BACnet dataset” folder contains files related to the dataset, detailed in Table 3. The “Controller simulation codes” folder includes Python codes for simulating normal behaviour and attacks:

• Chiller_Controller_Falsifying_Attack_Traffic: Simulation of the Falsifying attack on the chiller controller
• Chiller_Controller_Modifying_Attack_Traffic: Simulation of the Modifying attack on the chiller controller
• Chiller_Controller_Normal_Traffic: Simulation of normal chiller traffic
• Simulation_of_covert_channel_attack_with_EncryptsMassage_Chiller: Simulation of Covert Channel Attacks (message is encrypted) in Chiller Controller
• Simulation_of_covert_channel_attack_with_Hash_Massage_Chiller: Simulation of Covert Channel Attacks (message is hashed) in Chiller Controller
• Simulation_of_covert_channel_attack_with_plaintext_Chiller: Simulation of Covert Channel Attacks (message is plain text) in Chiller Controller
• VAVBOX_Controller_Normal_Traffic: Simulation of the control unit of the VAVBOX
• Weather_station_Controller_Normal_Traffic: Simulation of the control unit of the weather station
• AHU_Controller_Normal_Traffic: Simulation of the control unit of the air handling device

## 4 Experimental Design, Materials and Methods

**Fig. 1:**
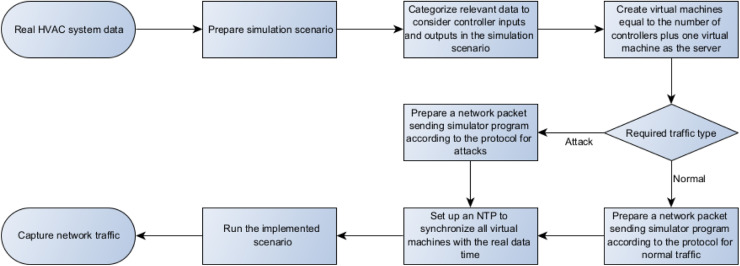
Proposed method framework.

In this paper, we propose a framework for converting building management data to BACnet protocol network data, providing data scientists and network security researchers with a suitable dataset for normal traffic and various attack traffic. Fig. 1 shows the proposed framework.

**Fig. 2:**
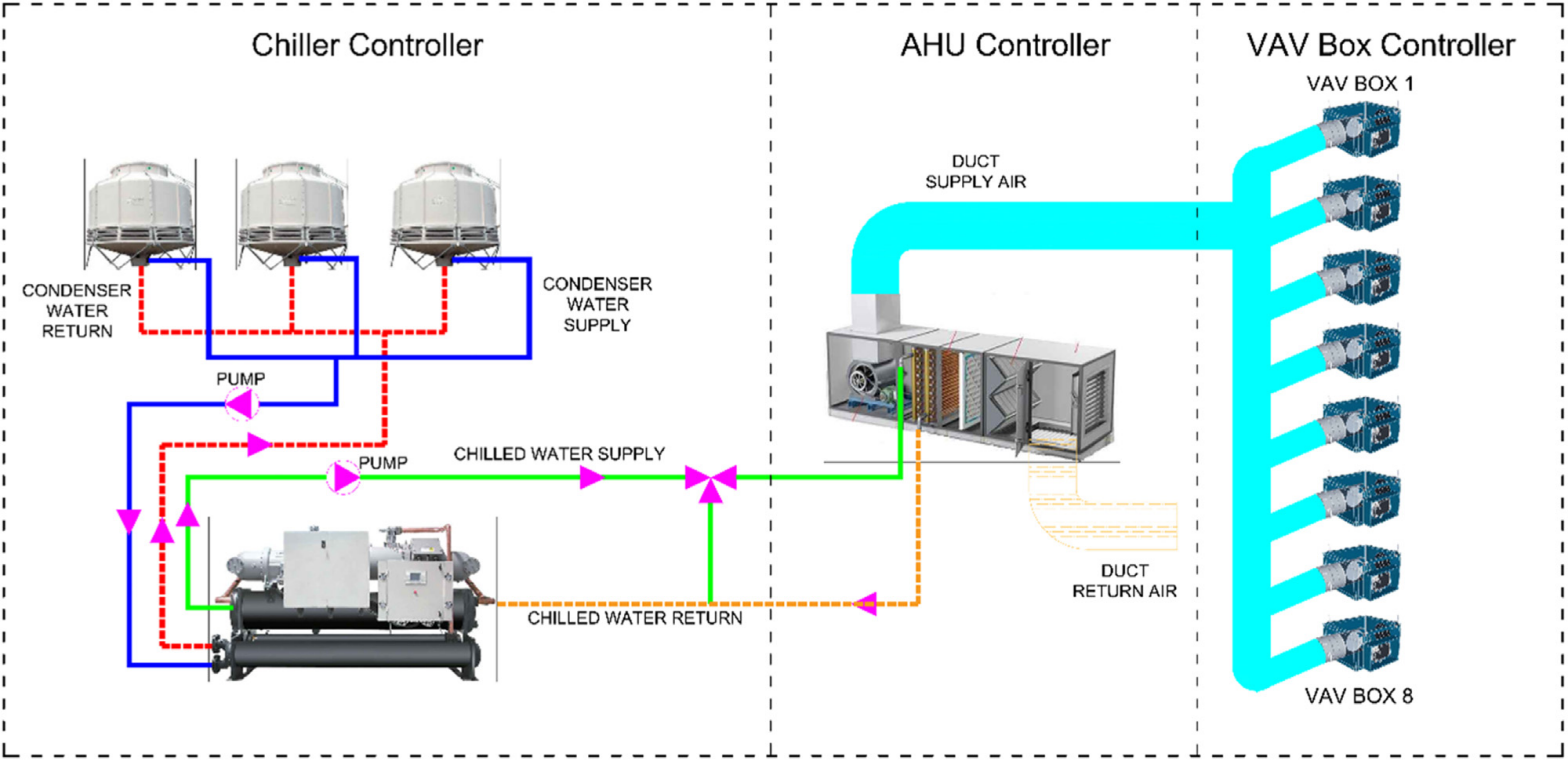
Proposed scenario.

To create the BACnet protocol traffic dataset, we used data from the Building and Energy Management System of Tampines College Global Campus in Southeast Asia, Singapore [1]. Based on the real HVAC system data, we created a communication network scenario as shown in Fig. 2.

The proposed scenario includes part of the existing data. Given the large number of equipment at Tampines campus, some of this equipment was selected as a representative of the HVAC system behaviour. In this selection, the complete cycle of HVAC operations was considered, and the behaviour of each component and its logical connection with other components was included. Essentially, we can assume that we evaluate the behaviour of a zone of the communication network as a representative of the entire network.

We implemented the plan by creating five virtual machines on a physical server. Four of these machines used the Raspberry Pi operating system due to its lower resource requirements compared to Windows or other common Linux operating systems. Table 1 shows the features used in the simulated controllers. Table 2 presents sample summary statistics for selected parameters from the collected data (as referenced in [1]) over the time period from March 10, 2012, to March 20, 2012, including the mean, median, minimum, and maximum values for parameters such as temperature and airflow.

**Table 1:** Features used in simulation.

Simulated controller	Period (Second)	Features	Unit
Chiller	180	Chilled Water Flow rate Chilled Water Return Temperature Chilled Water Supply Temperature Condenser Water Flow rate Condenser Water Return Temperature Condenser Water Supply Temperature Cooling Tons Efficiency Power Cooling Tower1∼3 Water Supply Temp. Chilled Water Pump On Off Status Condenser Water Pump On Off Status On Off Status	Cubic Feet Per Minute Degrees Celsius Degrees Celsius Cubic Feet Per Minute Degrees Celsius Degrees Celsius Tons Refrigeration Percent Kilowatt Hours Degrees Celsius - - -
Air Handling Unit	600	CO2Sensor Humidity Off Coil Temperature Static Pressure Supply Air Temperature On Off Status	Parts Per Million Percent Degrees Celsius Pascals Degrees Celsius -
VAV Box	600	Zone1∼8 Airflow Zone1∼8 Temperature	Cubic Feet Per Minute Degrees Celsius
Weather station	180	Humidity Wet bulb temperature Outdoor temperature	Percent Degrees Celsius Degrees Celsius

**Table 2:** Sample summary statistics parameters.

System Component	Parameter	Mean	Median	Min	Max
Chiller	Flow Rate (L/min)	300.5	295.0	200.0	450.0
Air Handling Unit	Temperature (C°)	22.3	22.0	18.0	27.0
VAV Box	Airflow (CFM)	350.7	340.0	150.0	500.0
Weather station	Humidity (%)	55.2	55.0	30.0	70.0

Since our goal was to generate real-like network traffic to create a dataset that reflects the real behaviour of the system, preprocessing was avoided to prevent the loss of critical information about the system's behaviour. However, to approximate real behaviour, some important data, particularly about the on/off status of equipment, were added to the dataset as follows:

• For the on/off status of the air handling unit, if the static pressure was greater than 50 Pascals, it indicated the fan was on (on status); for lower values, it was off.
• For the on/off status of the chiller's chilled pump, if the chilled water flow was greater than 0, the status was on; if it was 0, the status was off.
• For the on/off status of the condenser pump, if the condenser water flow rate was greater than 0, the status was on; if it was 0, the status was off.
• For the on/off status of the chiller, if the power was greater than 0, the chiller was on; if it was 0, the chiller was off.

Each of these virtual machines represents a set of equipment in the HVAC system, as shown in Fig. 2. On each of these virtual machines, Python code simulating the BACnet protocol HVAC controller was implemented. Each virtual machine has a unique BACnet protocol ID and an associated IP within the network IP range. The fifth virtual machine, running Windows 10, was used as the server.

The distribution of the simulated virtual machines is as follows:

• Virtual machine number 1 includes the air handling unit, which produces and regulates the air required for the HVAC system.
• Virtual machine number 2 includes the chiller, cooling towers, and pumps, which produce and transfer the chilled water needed for cooling.
• Virtual machine number 3 includes the VAV boxes, which distribute and regulate the final room temperatures.
• Finally, virtual machine number 4 is a weather station that assists other subsystems in better regulation.

Virtual machine number 5 acts as the server, receiving traffic from virtual machines 1, 2, 3, and 4. This virtual machine uses a BACnet protocol browser called Yabe. Additionally, Wireshark software is installed on this machine to capture and store network traffic.

**Fig. 3:**
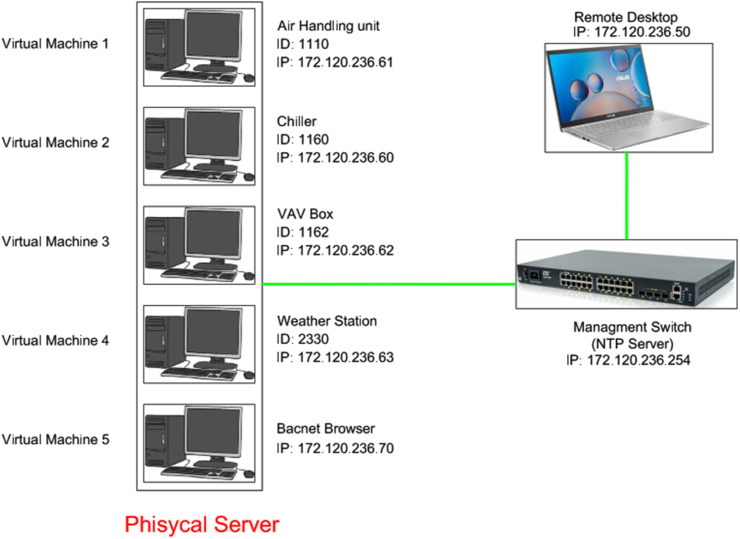
Network design for proposed scenario implementation.

To ensure greater accuracy and the potential for researchers to use other time-dependent information, the time on all servers 1was synchronized with the real-time reported in the actual dataset. The time for all virtual machines was synchronized with the management switch acting as the NTP server, which serves as the intermediary between the server and the remote computer (Fig. 3).

a. Normal Traffic
  To capture normal BACnet protocol traffic, the relevant data from each virtual machine was injected into the network as BACnet packets according to Table 3. Simultaneously, the entire network traffic was sniffed using Wireshark. This dataset can be used for training systems to recognize normal network behaviour.

**Table 3:** Captured traffic files.

File Name	Type	Description	Number of entries
Normal-traffic	Normal	Normal BACnet traffic from Mar 10, 2012, 00:00:00 to Mar 12, 2012, 06:19:25	24381867
CCA-plaintext	Normal & Covert Channel Attack (message is plain text)	Covert Channel Attack BACnet traffic from Mar 12, 2012, 02:06:23 to Mar 12, 2012, 12:00:23 and Normal BACnet traffic from Mar 12, 2012, 12:00:23 to Mar 13, 2012, 01:07:54	6445739
CCA-hash	Normal & Covert Channel Attack (text message is hashed)	Covert Channel Attack BACnet traffic from Mar 13, 2012, 01:47:48 to Mar 14, 2012, 03:17:48 and Normal BACnet traffic from Mar 14, 2012, 03:17:48 to Mar 15, 2012, 08:19:29	15280179
CCA-encryption	Normal & Covert Channel Attack (text message is encryption)	Covert Channel Attack BACnet traffic from Mar 15, 2012, 08:48:33 to Mar 15, 2012, 21:33:33 and Normal BACnet traffic from Mar 15, 2012, 21:33:33 to Mar 17, 2012, 06:26:13	12783439
Falsifying-in-chiller	Falsifying Attack	Falsifying Attack BACnet traffic from Mar 17, 2012, 06:39:50 to Mar 18, 2012, 01:19:17	5221777
Modifying-in-chiller	Modifying Attack	Modifying Attack BACnet traffic from Mar 18, 2012, 01:30:19 to Mar 19, 2012, 02:01:19	6871879
b. Falsifying and Modifying Attacks
  For simulating these attacks, we designed two types of attacks on the chiller virtual machine.

Falsifying Attack: In this attack, a random value between 4 and 5 degrees Celsius was added to the Chilled Water Supply Temperature feature of the chiller controller.

Modifying Attack: This simulation was performed on the chiller controller. In this attack, if the chilled and condenser pumps were on, they were reported as off, and if they were off, they were reported as on.

a. Covert Channel Attacks
  To simulate covert channel attacks, we used timing discrepancies in data transmission. The hidden message ("Hello Bob I am Alice") was converted to ASCII code, and based on whether the ASCII bit was 0 or 1, the transmission time of the chiller controller packets (all features) was reduced or increased by one second.

For better analysis by researchers using this dataset, the hidden message was sent once in plain text, once hashed using SHA3-256, and once encrypted using AES-256 at different time intervals through the chiller virtual machine.

The dataset and framework presented in this study can support both fault detection and cryptographic security within BAS. By utilizing real data combined with various simulated attack scenarios, this dataset enables researchers to test fault detection algorithms under realistic conditions, evaluating their robustness in identifying network performance issues resulting from potential attacks or faults. Additionally, the simulated attack scenarios, such as data manipulation and covert channel exploits, highlight specific vulnerabilities within the BACnet protocol that could be mitigated through cryptographic measures. Testing encryption techniques on this dataset allows researchers to assess how well these techniques protect BACnet communications, ensure data integrity, and safeguard sensitive information within smart building environments.

This study presents a dataset framework specifically designed to address challenges within BAS, particularly in relation to the BACnet protocol. The current dataset effectively simulates various cyber-attack scenarios, yet future efforts will focus on increasing data diversity and adaptability by incorporating real-world data from Software-Defined Networking (SDN) environments that support BACnet. Expanding this framework to SDN-based architectures will enable the simulation of practical attack scenarios in smart building environments, providing a broader applicability across diverse BAS environments and allowing for the assessment of novel security threats. Standard BACnet attacks, such as data spoofing, data manipulation, and covert channel attacks, could also be executed within SDN environments to evaluate their impact on SDN-integrated building automation systems. Moreover, simulating emerging SDN-specific attack vectors, such as data path rerouting and controller compromise, could yield further insights into the security requirements of BACnet within contemporary network infrastructures. Integrating cryptographic protocols within this framework may also add additional layers of protection for sensitive control systems, supporting real-time fault detection and anomaly management, ultimately enhancing the resilience of smart building networks.

## Limitations

Not applicable.

## Ethics Statement

The authors confirm that they have read and follow the ethical requirements for publication in Data in Brief. The current work does not involve human subjects, animal experiments, or any data collected from social media platforms.

## Credit Author Statement

**Seyed Amirhossein Moosavi:** Conceptualization, Methodology, Data Curation, Writing - Original Draft, Visualization. **Mojtaba Asgari**: Supervision, Project Administration, Writing - Review & Editing. **Seyed Reza Kamel**: Research Consultant, Validation, Writing - Review & Editing.

## Declaration of the Use of AI-Assisted Technologies in Scientific Writing

The authors declare that they have assisted ChatGPT-4 in writing process of the paper for only translating the article from Persian to English language not for any other purposes.

## Data Availability

KaggleBACnet network dataset (Original data).
